# The greater snow goose *Anser caerulescens atlanticus*: Managing an overabundant population

**DOI:** 10.1007/s13280-016-0887-1

**Published:** 2017-02-18

**Authors:** Josée Lefebvre, Gilles Gauthier, Jean-François Giroux, Austin Reed, Eric T. Reed, Luc Bélanger

**Affiliations:** 1 Canadian Wildlife Service, Environment and Climate Change Canada, 801-1550 Avenue d’Estimauville, Québec, QC G1J 0C3 Canada; 2 Département de Biologie & Centre d’Études Nordiques, Université Laval, 1045 Avenue de la Médecine, Québec, QC G1V 0A6 Canada; 3 Département des Sciences Biologiques, Université du Québec à Montréal, Station Centre-ville, P.O. Box 8888, Montréal, QC H3C 3P8 Canada; 4 Canadian Wildlife Service, 1933 Place Michel-Valcourt, Québec, QC G2B 1Y2 Canada; 5 Canadian Wildlife Service, Environment and Climate Change Canada, 5019 - 52nd Street, Yellowknife, NT X1A 2P7 Canada

**Keywords:** *Anser caerulescens atlanticus*, Greater snow goose, Management, North America, Overabundant population

## Abstract

Between the early 1900s and the 1990s, the greater snow goose *Anser caerulescens atlanticus* population grew from 3000 individuals to more than 700 000. Because of concerns about Arctic degradation of natural habitats through overgrazing, a working group recommended the stabilization of the population. Declared overabundant in 1998, special management actions were then implemented in Canada and the United States. Meanwhile, a cost–benefit socioeconomic analysis was performed to set a target population size. Discussions aiming towards attaining a common vision were undertaken with stakeholders at multiple levels. The implemented measures have had varying success; but population size has been generally stable since 1999. To be effective and meet social acceptance, management actions must have a scientific basis, result from a consensus among stakeholders, and include an efficient monitoring programme. In this paper, historical changes in population size and management decisions along with past and current challenges encountered are discussed.

## Introduction

**Fig. 1:**
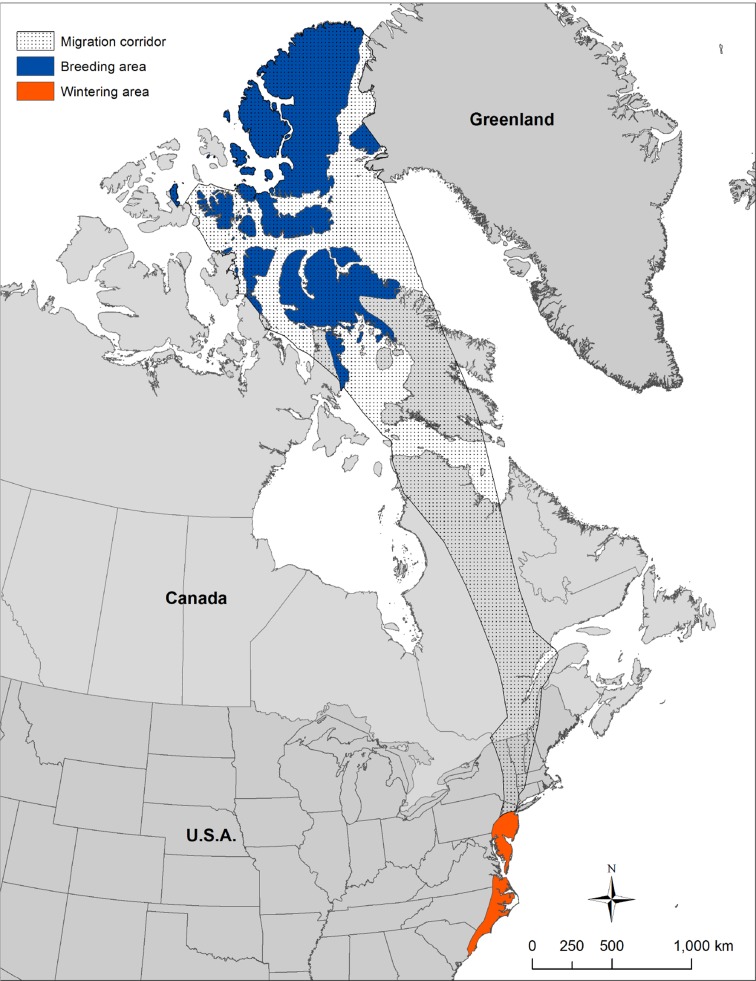
Range map of greater snow geese in North America

The snow goose *Anser caerulescens* is one of the two most abundant goose species in North America and is distributed across the whole continent. It includes two sub-species: the greater snow *Anser c. atlanticus* and the lesser snow goose *Anser c. caerulescens* (Canadian Wildlife Service Waterfowl Committee 2015). This paper focuses on the greater snow goose which is largely confined to the Atlantic Flyway and constitutes a single population (Fig. 1). It breeds in the northern and eastern parts of the Canadian Arctic Archipelago and northwestern Greenland and winters along the U.S. Atlantic coast from New Jersey to North Carolina (Mowbray et al. 2000). We also emphasize management issues from a Canadian perspective, though the U.S. situation is addressed as needed.

In recent decades, North American goose managers have faced a new problem of overabundant populations. Some species have escaped from natural regulatory processes, which result in the overuse of habitats and their subsequent degradation. This can have negative or positive impact on other species but may ultimately result in a reduction of local biodiversity (Côté et al. 2004; Jefferies et al. 2006; Bråthen et al. 2007). Overabundant geese often benefit from anthropogenic activities by adapting their feeding behaviour to agricultural changes, which can lead to crop depredation issues (Jefferies et al. 2004; Abraham et al. 2005). On the other hand, their abundance can also generate economic benefits through bird watching and hunting (Groupe Conseil Genivar Inc. 2005; U.S. Department of the Interior et al. 2011). High abundance levels can therefore generate conflicts between people who benefit from the presence of these populations and those who suffer economic losses. The demographic explosion of snow geese, due to a combination of anthropogenic and natural causes (Gauthier et al. 2005), is a prominent example of a wildlife population considered overabundant in North America. This has brought exceptional management measures that have had various degrees of success. In this paper, we review historical changes that occurred in the greater snow goose populations, decisions taken to manage this overabundant population over the past 20 years, the monitoring programmes put in place to evaluate the success of the management actions, and the major challenges encountered and lessons learned throughout the entire process.

## Historical population changes

At the beginning of the twentieth century, the population of greater snow geese was estimated at around 3000 individuals (White and Lewis 1937; Lemieux 1959). Subsequently, the size of the population changed in response to various management actions, but overall exhibited a prolonged period of increase as detailed below.

### Initial protection through the creation of protected areas

Between 1934 and 1967, 12 wildlife refuges (>55 000 ha) were created in the U.S. wintering areas to provide resting areas for several waterfowl species, including greater snow geese, with an additional refuge of 38 000 ha created in 1990 in North Carolina (Gauthier et al. 2005). In southern Québec, where snow geese stop during their spring and fall migration, the Canadian Wildlife Service (CWS) created the Cap Tourmente National Wildlife Area in 1978, a traditional stopover area for geese. Several Migratory Bird Sanctuaries were also established along the St. Lawrence River during the 1970s and 1980s. The total protected areas in southern Québec represent about one tenth of the area of those on the wintering grounds (Gauthier et al. 2005). These protected areas, where hunting was prohibited or controlled, were intensively used by geese as they were mostly located near agriculture lands where they could exploit an abundant source of food. These conditions contributed to the initial increase of the population (Abraham and Jefferies 1997; Reed et al. 1998).

### Liberalization of hunting

**Fig. 2:**
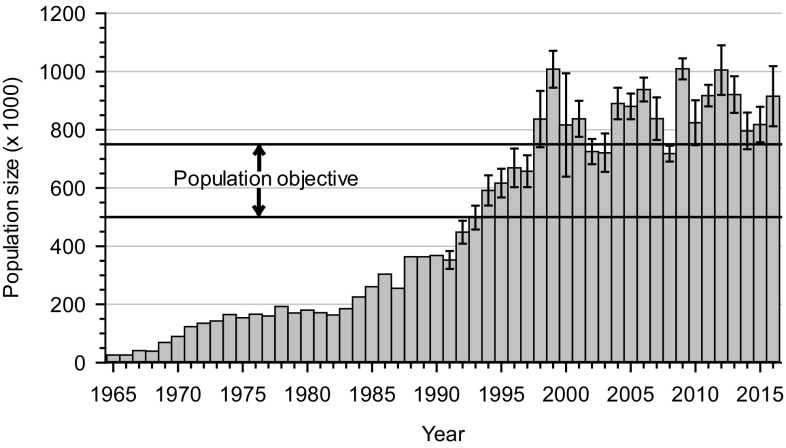
Greater snow goose population size determined during the spring survey, 1965–2015. *Error bars* are 95% confidence intervals (see Béchet et al. 2004b for methodological details)

Up to the 1960s, the small size (<40 000 individuals) of the population was a cause of concern because of potential risk from accidental oil contamination from adjacent seaports where geese were concentrated during some periods of the year (Anonymous 1981). In Canada, fall sport hunting was, nevertheless, allowed during this period because of the restricted area and short period during which Canadian hunters had access to the birds. However, hunting was closed in the U.S. in 1931 and only reopened in 1975 after the population had increased to well over 100 000 birds (Reed 1990). This was followed by a period of about ten years when population estimates remained relatively stable (Fig. 2). The combined U.S. and Canadian harvest was apparently sufficient to maintain the population at a stable level, mainly through a reduction in adult survival, the parameter to which population growth is most sensitive (Table 1; Gauthier and Brault 1998; Menu et al. 2002).

**Table 1:** Summary of estimated harvest rate of adult greater snow geese by time period and general population trend during these periods from 1974 to 2014 (updated from Calvert et al. 2007)

Period	Adult harvest rate (%)	Population trends
1975–1984	11.5	Stable
1985–1997	6.1	Increasing
1998–2002^a^	12.9	Declining
2003–2007^a^	8. 6	Increasing
2008–2011^b^	11.0	Stable
2012–2014^b^	13.5	Possibly declining

^a^Special conservation measures in Canada

^b^Conservation Order in the U.S. and special conservation measures in Canada

### Rapid growth of the population

In the 1980s, the core area of the greater snow geese winter distribution changed as increasing numbers of birds short-stopped along their migration corridor to overwinter to the north of the previous traditional range (Calvert et al. 2005). Warming temperatures in winter along with an increase in corn *Zea mays* production, an important food source contributed to this shift in distribution (Gauthier et al. 2005). An important consequence, however, is that this change in distribution led to a decrease in hunting mortality in winter as exposure to hunters was reduced.

In southern Québec, a gradual shift in the distribution of geese during their staging period also took place. Formerly confined to the bulrush *Schoenoplectus americanus* (formerly known as *Scirpus americanus*) marshes of a short section of the St. Lawrence estuary, geese expanded their distribution all along the St. Lawrence River, predominantly to farmlands devoted to small cereals, corn, and hay crops (Reed et al. 1998; Gauthier et al. 2005). Although this expansion could have theoretically increased hunting opportunities, it occurred in areas with no previous tradition of recreational harvest of snow goose. Moreover, geese tended to move in very large flocks in those newly occupied areas and exhibited unpredictable and long-distance movements between roosting areas along the river and feeding sites on farmlands, rendering hunting more challenging (Béchet et al. 2010). Thus, this change in behaviour further contributed to reductions in hunting pressure during that time.

Despite a continuous liberalization of daily and possession bag limits starting in the 1980s, average annual population growth reached 9% between 1983 and 1997 (Reed et al. 1998). During that period, goose harvest increased in Canada but not in the U.S. resulting in a decline of hunting mortality overall (Table 1), which led to a doubling of population size every eight years (Gauthier and Brault 1998).

## Consequences and concerns linked to population increase

### Natural habitats

Destruction of coastal saltwater habitats by lesser snow geese due to overgrazing and grubbing has been well described in the Arctic (Abraham and Jefferies 1997; Jefferies et al. 2004). However, greater snow geese use primarily freshwater habitats in Arctic Canada and their impacts in those habitats are not as severe (Gauthier et al. 2006). Studies conducted in the 1990s at the largest greater snow goose breeding colony on Bylot Island, Nunavut, Canada, showed that annual plant production and specific composition were reduced by goose grazing even though the total consumption by geese in wetlands represented only 46% of the estimated carrying capacity of the potential foraging habitat of the island (Gauthier et al. 1995, 2004; Massé et al. 2001). Nevertheless, there was some concern that this rapidly increasing population may soon exceed the carrying capacity of their breeding areas if no actions were undertaken (Giroux et al. 1998a).

In the 1980s and 1990s, studies were conducted on the interaction between snow geese and their traditional bulrush marsh habitat used during fall and spring staging in southern Québec. They revealed a decreasing number of goose-days in some marshes due to a decline in plant productivity, a change in floristic composition, and an increase of marsh erosion, all of which were partially caused by the geese (Giroux and Bédard 1987; Giroux et al. 1998b; Lefebvre et al. 2001). This suggested that the carrying capacity of those habitats may have been reached, especially in bird sanctuaries. Nonetheless, marshes denuded of vegetation resulting from goose feeding were not observed (Giroux et al. 1998b).

In winter, greater snow geese traditionally used coastal salt marshes dominated by cord grass *Spartina alterniflora*. Their grazing impact was considered negligible until the 1960s, but with the increase of the population in the 1970s and 1980s, sections of marshes heavily used by geese in some wildlife refuges became completely denuded (Smith and Odum 1981; Giroux et al. 1998b). Even if this impact was very limited in comparison to the total area of salt marshes, it was still significant locally and could have both positive and negative effects on other wildlife species (Giroux et al. 1998b).

### Impact on farmlands

Starting in the 1980s, the growing use of farmlands by geese during winter in the U.S. and spring staging in Québec began to cause crop damage (Gauthier et al. 2005). Such damage is assessed and compensated within Canada but not in the U.S. In Québec, most damage occurs in spring soon after snow melts in hayfields when young shoots of grass and legumes start to grow (Bédard et al. 1986; Filion et al. 1998). The expansion in the distribution of geese in Québec in the 1990s occurred mostly in corn-growing areas where geese mostly fed on waste grain (Giroux and Bergeron 1996). However, when spring is early and corn sown before the departure of geese, they can pull up newly sprouting shoots, resulting in significant local damage. A compensation programme for goose damage has been in place since 1992 in Québec, and is funded by budgets allocated to the agricultural department by both federal and provincial governments (Filion et al. 1998). Compensations cover only 80% of losses, and farmers must thus bear some economic losses due to the presence of geese. Damage is determined regionally in each agricultural zone by comparing yields in field plots exposed and non-exposed (by exclosures) to goose grazing (Filion et al. 1998). Between 1992 and 2015, the annual amount paid averaged US$603 000 and varied between US$125 500 and US$1 761 500 (Dubé, pers. comm.). In 2016, compensation was raised to cover 90% of losses.

## Management decisions linked to the overabundance problem

In the U.S. and Canada, responsibility for the protection and conservation of geese and other migratory birds comes under the authority of the two federal governments, as established by the Migratory Birds Convention Act in Canada and the Migratory Bird Treaty Act in the U.S. In Canada, the Act is implemented by the CWS of Environment and Climate Change Canada (ECCC) while hunting activities are co-managed with the provinces. The U.S. Act authorizes the United States Fish and Wildlife Service (USFWS) to set general hunting regulations annually, but each state establishes the dates of its own individual hunting seasons. While state regulations may be more restrictive than the federal ones, they cannot be more liberal. The USFWS and individual states share the responsibility of enforcing hunting regulations to protect migratory birds.

### Administrative flyways

In North America, waterfowl are managed through four administrative flyways, from east to west, the Atlantic, Mississippi, Central, and Pacific Flyways (Anderson and Padding 2015). Each flyway is led by a Council composed of one member from each represented state and province. The Atlantic Flyway Council, which encompasses most of the greater snow goose’s range, was created in 1952 and the eastern Canadian provinces joined in 1958 (Addy and Kennedy 1969; Hawkins et al. 1984). Each Flyway Council is advised by a Technical Committee of biologists from each state and province and from the USFWS and CWS. At least two meetings are scheduled each year to share information, develop monitoring programmes, and to provide advice to both federal agencies about harvest regulations (Anderson and Padding 2015). Each country is independent and can implement its own regulation. However, the Flyway Council provides a permanent process of consultation and collaboration among members when implementing new actions and regulations.

### Mechanisms to set hunting regulations

In Canada, the schedule for developing hunting regulations begins in November of a given year and results the following June in the publication of regulatory amendments in the *Canada Gazette*, the official newspaper of the Government of Canada. These amendments are, in effect, for the hunting season for the year of publication and for overabundant species (snow goose), for the following spring. As of 2014, the Canadian Migratory Bird Regulations for game birds are being revised every two years. The process mainly involves consultation by CWS biologists of stakeholders through a formal process, followed by a broader consultation period opened to all Canadians, who can express their opinions about the proposed regulatory amendments. A special committee was further established with stakeholders to deal with the management issues specific to the Greater Snow Goose in Québec, the Technical Committee on the Integrated Management of Greater Snow Geese, as detailed below.

In the U.S., the USFWS starts the consultation process in late January and publishes a series of documents in the Federal Register that describes proposed hunting regulations. The four Flyway Councils are consulted several times throughout the annual cycle, and the public has the opportunity to express comments on the proposed regulations. Final regulations are published in late September and take effect the same year. A hunting guide is published by each state to inform their hunters about their specific regulations. Starting in 2016, a new regulatory schedule is being put in place to allow more time for discussion, an extended review process as well as a longer period for public consultation (Padding, pers. comm.).

### Working groups and the scientific evaluation of the overabundance problem

In 1996, a working group composed of scientists and managers was formed, the *Arctic Goose Habitat Working Group.* The Committee’s mandate was to conduct a rigorous scientific evaluation of the snow goose problem across the continent, to increase awareness regarding this situation among all stakeholders (government, non-governmental organizations, and general public) and to propose solutions. This group published a first report in 1997 (Batt 1997) which primarily addressed the situation regarding the lesser snow goose.

In 1997, a sub-committee was formed to assess the particular case of the greater snow goose, reported in Batt (1998). Their main conclusions were that (1) the population was doubling every eight years based on the prevailing conditions, (2) the use of new habitats such as farmlands was likely to continue to increase, (3) the carrying capacity of several natural habitats was reached, or would be soon, which would likely lead to significant negative impact on those habitats, other species, and the geese themselves, and (4) the economic losses in farmlands due to the presence of geese would continue to increase. The committee then recommended that the population should be stabilized between 800 000 and 1 000 000 birds by 2002 (Giroux et al. 1998a). This laid the foundation for the management of this population in future years.

### Management actions

Publication of the report by Batt (1998) quickly led authorities to declare greater snow geese as overabundant and allowed the use of special conservation measures in Canada starting in 1999. These included the legalization of formerly prohibited hunting techniques, such as sneaking (stalking) on goose flocks, use of electronic goose calls, baiting to lure birds (under specific permits and conditions), and a spring conservation harvest. It was argued that the spring harvest could be considered a conservation strategy to protect the goose habitats. This last measure was by far the most significant one and a first in North America since the signing of the Migratory Bird Convention in 1916, which specified no hunting of migratory game birds between 10 March and 1 September. The spring conservation harvest aimed to increase overall hunting mortality, primarily on adults, and was authorized only on farmlands to attenuate goose damage to crops at that time (Calvert et al. 2007). An animal rights organization took the Canadian Government to court over the decision to declare snow geese as overabundant but a rapid ruling was made in favour of the Canadian government (Animal Alliance of Canada vs. Canada (Attorney General) (T.D.), [1999] 4 F.C. 72). In the U.S., a similar legal challenge delayed the adoption of conservation actions until 2009 when a special harvest using more permissive rules could be finally implemented under the name *Conservation Order*.

At the provincial level in Québec, the Technical Committee on Integrated Management of Greater Snow Geese was established in 1996. This consisted of governmental and non-governmental agency representatives involved in the management of this population and different stakeholders, including representatives of hunters, outfitters, birdwatchers, farmers, tourist associations, conservation groups, provincial and federal departments of wildlife and agriculture, and university researchers. Representatives of these organizations still meet annually to share current information about the greater snow goose population and to discuss their respective concerns. This turned out to be an important forum to exchange information and to seek consensus regarding management objectives and actions required to reach them. Consultation with other groups such as the Inuit hunter community was more limited and mostly involved informal meetings during visits of biologists to northern communities.

The CWS published Action Plans for the 1997–2002 and 2005–2010 periods with the main objectives of preventing damage to natural snow goose habitats and reducing crop damage while maintaining the economic benefits associated with the passage of migrating geese in Québec and improving the long-term management of the population. In order to facilitate the coordination and enhance the participation of partners, a workshop was organized in 2012 with all stakeholders to prepare the next action plan. Following this workshop, an updated Action Plan (2013–2018; Anonymous 2013) was published and implemented.

The CWS Action Plans established a target spring population between 500 000 and 750 000 (Bélanger and Lefebvre 2006). This was based on a cost–benefit analysis of selected management scenarios based on socio-economic values integrated into a single index linked to the population size observed between 1965 and 2004 (see full explanation in Table 2). Identifying the potential indicators of socio-economic values associated with the presence of geese throughout their annual cycle (hunting, birdwatching, refuge public attendance, crop damage, etc.) was the first objective. The index was obtained from principal component analyses performed on the various socio-economic value indicators in relation to the presence of geese on their wintering grounds, staging areas, and from a continental standpoint. Knowledge of the carrying capacity and ecological integrity of natural habitats, which the birds exploited throughout their annual cycle, as well as the potential of sport hunting to act as a means of population control were also taken into account to determine the optimal management scenario. Subsequently, the Atlantic Flyway Technical Committee adopted the same population objective in their management plan (Snow Goose, Brant, and Swan Committee of the Atlantic Flyway Gamebird Technical Section 2009). Several long-term monitoring programmes were utilized to assess the success of the management actions taken to achieve this goal.

**Table 2:** Summary of ecological, management, and socio-economic issues associated with various sizes of the greater snow goose population throughout its annual cycle based on the reviews of Batt (1998) and Reed and Calvert (2007) and the analysis of Bélanger (unpubl.), and the status assigned to various population levels for management purpose

Population size	Population status	Use of farmlands versus natural habitat in relation to carrying capacity (K)	Socio-economic values^a^	Hunting and population control
0–250 000 geese	Historical level population	Wetland ≥ Farmlands Arctic breeding habitats < K Migration & wintering natural habitats < K	Benefits = Costs Localized benefits and low crop damage	Restrictive regulations
250 000–500 000 geese	Abundant population	Farmlands > Natural Arctic breeding habitats < K Migration & wintering natural habitats ≈ K	Benefits > Costs Widespread benefits and moderate crop damage	Standard to liberal regulations
500 000–750 000 geese^b^	Very abundant population	Farmlands ≫ Natural Arctic breeding habitats < K Migration & wintering natural habitats > K	Benefits ≫ Costs Very high benefits and high crop damage	Liberal regulations
750 000–1 000 000 geese	Over abundant population	Farmlands ≫ Natural Arctic breeding habitats < K Migration & wintering natural habitats > K	Benefits > Costs Saturation of benefits and very high crop damages	Liberal regulations and special conservation measures (spring harvest)

^a^Consider all socio-economic benefits related to the presence of geese including activities such as hunting, bird watching, tourism, etc

^b^Current population objective

## Monitoring programmes

### Annual spring survey

An aerial survey of the population has been conducted every spring by CWS since 1965. During approximately three weeks in spring, the whole population gathers in southern Québec in a relatively limited area (Béchet et al. 2004a). This makes it possible to obtain a more accurate count-based estimate than at any other time of the year, when the population is much more dispersed.

During the survey, photographs are taken of all of goose flocks found. The estimated size of population is based on a two-stage, combined stratified ratio estimator using partial counts and visual estimates of photograph flocks in different size classes (see Béchet et al. 2004b for more details). The high concentration of birds combined with their size and white colour, which contrasts well against a dark background, makes it possible to conduct a comprehensive survey of the entire population. Capture–recapture techniques based on radio-marked birds were used in 1998–2000 to estimate the proportion of birds missed during the aerial surveys (Béchet et al. 2004b). Following recommendations from this study, some modifications were made to the survey in 2004 to cope with the increasing dispersion of geese throughout the staging area. Most importantly, the number of aircraft was increased from one to five to cover the entire area (22 000 km^2^) within a single day (Calvert et al. 2007).

### Monitoring of reproduction, habitat, and goose banding

Greater snow goose reproductive success has been monitored by Université Laval in collaboration with CWS at the Bylot Island breeding colony, Nunavut (73°08′N, 80°00′W), annually since 1989. Breeding geese are concentrated over a 40 km^2^ area where nesting density averages 400 nests/km^2^ (Gauthier et al. 2013). Breeding propensity, nest density, nesting phenology, clutch size, nesting success, and production of young at the end of the summer are measured. In addition, annual primary production and goose grazing impact upon wetlands are monitored annually in the nesting colony as well as in the most important brood-rearing areas (Valéry et al. 2010; Gauthier et al. 2013).

Aerial surveys were conducted every five years from 1983 to 2008 to obtain an estimate of the size of the largest breeding colony in the Canadian Arctic and to document changes over time (Reed and Chagnon 1987; Reed et al. 1992, 2002). These surveys were conducted during the brood rearing period and were based on sample plots, stratified in relation to habitat suitability (Reed et al. 2002).

At the end of summer, moulting adults and young are captured and banded annually (over 93 000 birds banded to date) and a sample of adult females is marked with a coded plastic neck band. Young are also measured and weighed to determine their size and monitor their growth. Banding data are integrated into the North American Bird Banding Laboratory database, jointly operated by the U.S. Geological Survey and the CWS, and which also receives band numbers from shot birds reported by hunters.

Finally, annual productivity has been estimated annually by ground surveys conducted on the main autumn staging areas in Québec since 1973, based upon the methodology of Lynch and Singleton (1964).

### Observation of neck-banded birds

To improve the estimation of demographic parameters such as survival rate, observations of neck-banded females, mostly reported by birders, have been collected throughout their annual cycle, but especially during the spring and fall staging periods in southern Québec (Gauthier et al. 2001). This programme, primarily based on females marked on Bylot Island, has been coordinated by Université Laval. Since 1990, 14 058 females have been neck-banded and the database includes > 76 000 resightings (Cadieux, pers. comm.).

### Monitoring of the harvest

Annual harvest of greater snow goose in Canada has been estimated since 1967 through a CWS national survey conducted among hunters. This survey has two components, the Harvest Questionnaire Survey and the Species Composition Survey, and both are conducted among a random sample of migratory bird hunting licence owners (Gendron and Smith 2015). Information on the size of the U.S. harvest has been available since the reopening of the hunt in 1975 through a similar survey among the U.S. hunters conducted by the USFWS, the Migratory Bird Harvest Information Program (Elden et al. 2002). Finally, a special survey was implemented in 1999 in Canada and 2009 in the U.S. to estimate the size of the harvest during the special conservation harvest in spring in Canada and the Conservation Order in the U.S.

## Effect of special conservation measures on the demography and behaviour

In 2007, an updated scientific evaluation of the greater snow goose population was produced to assess the effects of the special management measures implemented since 1999 to control the population (Reed and Calvert 2007). This report was based on the most up-to-date scientific studies carried out to monitor the impact of these actions on the population. It covered the period from 1965 to 2003, with a special emphasis on 1998–2003.

Population estimates during the first five years following the implementation of the special conservation measures in Québec (Fig. 2), which includes the spring conservation harvest, and the partial liberalization of hunting regulations in the U.S. indicated a stabilization of the population and even a possible declining trend (Calvert et al. 2007). This result was not associated with changes in the survey methods as most geese were still located in the traditional survey zone. This drastic change in population trend was largely due to a doubling of the harvest rate of adults (Table 1), which led to a significant decline in adult survival, from a mean of 83.0% from 1990 to 1997 to 72.5% from 1998 to 2002 (Calvert and Gauthier 2005). The spring conservation harvest in Québec was the management action that contributed the most to the increase in adult mortality during that period, although an increase in mortality during the winter period in the U.S. also contributed.

**Fig. 3:**
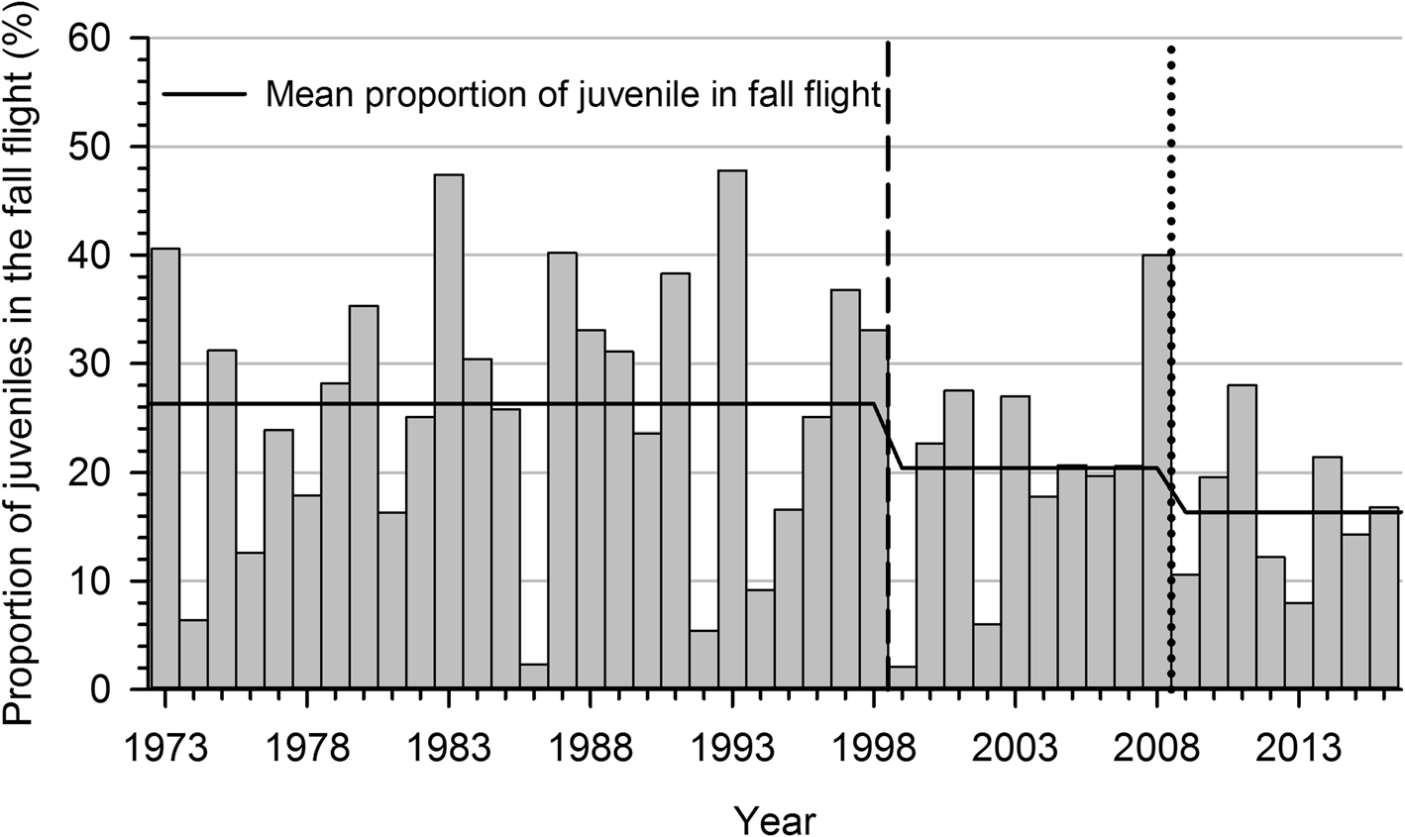
Proportion of juvenile greater snow geese during fall staging in southern Québec from 1973 to 2015. Data are from Reed et al. (1998) and Lefebvre (unpubl.). The *dashed line* indicates the start of special conservation measures in Canada and the *dotted* one the Conservation Order in the United States

Following the implementation of the spring conservation harvest, a reduction in the breeding propensity, a delay in laying date, and a decrease in clutch size were observed at the Bylot Island breeding colony (Mainguy et al. 2002; Bêty et al. 2003; Reed et al. 2004). The negative impact on reproduction was largely a consequence of an increase in spring disturbance caused by the spring conservation harvest, which led to an increase in energy expenditure, a reduction in food intake and ultimately to a reduced body condition at the end of the spring staging period (Féret et al. 2003; Béchet et al. 2004a). Morrissette et al. (2010) showed that the reduction in overall productivity of the population observed after 1998 (Fig. 3) was largely a carry-over effect of the spring harvest and not a density-dependent effect. This reduction in productivity contributed to the stabilization of the population during this period (Gauthier and Reed 2007).

Monitoring the impact of goose grazing on the tundra wetlands at the Bylot Island breeding colony indicated that the reduction in net aboveground primary production and aboveground biomass documented during the early 1990s (Gauthier et al. 1995) stopped following the implementation of the special conservation measures (Valéry et al. 2010). In most recent years, a significant increase in primary production has even been observed, but this is probably more a consequence of the climate warming than of the special conservation measures (Gauthier et al. 2013).

On the staging areas of the St. Lawrence estuary, there was no clear indication that these measures have affected the natural habitats, but monitoring has not been as regular here as in the Arctic. Nevertheless, bulrush primary production has remained stable in some marshes and slightly increased in others (Girard 2009). These changes may simply reflect the reduced use of some marshes by staging geese that now heavily depend on agricultural lands to feed. In the U.S., the total area of salt marshes that has been impacted by geese has not increased although monitoring has been even more limited than in Québec. The use of agricultural lands on the wintering grounds and the implementation of a controlled hunt in some refuges have also reduced the use of these marshes (Calvert et al. 2007).

The Canadian spring conservation harvest was only allowed in farmlands, in order to reduce crop damage, and not in natural habitats (marshes). Despite the added hunting activity, goose feeding in farmlands intensified. However, a greater dispersal of geese through the agricultural landscape was also observed, possibly due to hunting disturbance, which indirectly reduced crop damage in some localities (Calvert et al. 2007). Farmers also increased and coordinated their hazing activities at the same time, which also contributed to a reduction of crop damage in some areas. Overall, there was no direct relationship between the annual goose population size and crop damage across southern Québec. Other factors such as spring weather, agricultural practices, bird behaviour including their regional movements, and the intensity of hunting and scaring activities had confounding effects (Calvert et al. 2007).

Since 1999, greater snow goose population estimates have fluctuated between 700 000 and 1 000 000 birds (Fig. 2). Even though the current population remains above the population objective of 500 000 to 750 000 birds, we can claim that all the measures put in place to control this population over the past two decades have been successful because, if the growth rate that prevailed during the 1990s had been maintained, the population was projected to be as large as 3 million birds. Of course, density-dependent effects could have operated and slowed growth at some point. However, this is unlikely in the short term considering that the carrying capacity of the Arctic habitat had not yet been reached in the late 1990s (Massé et al. 2001) and may have actually increased since then due to climate warming (Gauthier et al. 2013). This was the case with the lesser snow goose population where no density-dependent effects were observed during this period (Leafloor et al. 2012).

## Discussion

### Biological challenges

The management of an overabundant species presents numerous challenges. For instance, the greater snow goose has shown a high potential to rapidly adapt to changing conditions. Their increased use of farmlands for feeding in response to large-scale changes in the agricultural landscape and their changes in behaviour in response to the spring conservation harvest are two obvious examples. Despite the relative stability of the population for almost two decades now, some of the environmental conditions that contributed to their rapid population increase in the late twentieth century still exist and may actually be increasing in intensity. These include climate warming, especially on arctic breeding grounds (Gauthier et al. 2013), and the continuous spread of corn both on the staging and wintering areas, a high-quality food for geese on farmlands that depend on grain market conditions. These factors have initially contributed to better body condition, higher reproductive success, and reduced natural mortality (Gauthier et al. 2005).

One could argue that the determination of a target population size should be primarily based on the carrying capacity of the natural habitats used by geese, but this is not easy to determine. At the principal breeding colony on Bylot Island, carrying capacity has not yet been reached (Massé et al. 2001). Recent nest surveys conducted after a 30-year gap at another breeding site on Ellesmere Island suggested a lower rate of population growth than that of the whole population (Lefebvre, unpubl.). Assessing the carrying capacity of staging and wintering areas is even more difficult due to the various habitats used by geese. If the current population was entirely restricted to natural habitats, clearly their carrying capacity would be exceeded, especially on the staging areas, but nowadays the bulk of goose feeding occurs in farmlands. However, integrating farmlands into the estimation of carrying capacity introduces the problem of social tolerance into any policy decision making. It is unrealistic and prejudicial to ask farmers to bear the cost of maintaining a large goose population for the benefits of other users such as hunters and birdwatchers. A drastic reduction of the goose population to levels that prevailed in the 1970s, when staging and wintering geese were largely confined to natural habitats, would not solve the problems either. It is highly unlikely that geese would stop using farmlands where they can feed on a high-quality food source. How much farmland habitats could or should be included in an estimation of the carrying capacity remains an unresolved question, especially when considering that farmers are in business to feed their livestock and ultimately humans, not geese.

### Management challenges

A great challenge for managers of an overabundant population is to obtain a consensus on management objectives among stakeholders (hunters, outfitters, farmers, birdwatchers, tourist industry), who often have very divergent interests. Maintaining the participation of these various groups and their long-term commitment to a common goal (i.e. management of this population) as well as the financial support of partners for various monitoring or mitigation programmes (e.g. goose scaring on sensitive crops or compensation schemes) are other major challenges. These problems become even more acute when management actions appear to be successful, as in the present case, even though the target population is not yet reached.

In an effort to determine a target population size (in terms of minimum and maximum population levels), a broader approach based on ecological and social considerations was favoured in the early 2000s. The conclusion was that a spring population of 500 000 to 750 000 birds represented an optimal ecological and social management scenario for greater snow geese in North America (Table 2; Bélanger and Lefebvre 2006; Bélanger et al. 2007). This level allows the maintenance of a healthy population, which would be resilient to potential natural or anthropogenic catastrophes, minimizes the risk of damage to the ecological integrity of natural habitats and associated biodiversity, limits crop damage to an acceptable level, and optimizes the socio-economic benefits related to the presence of geese. This approach was recently used in a population of pink-footed geese *Anser brachyrhynchus* (Madsen and Williams 2012).

### Lessons learned

Management of the greater snow goose population has become a major issue over the past three decades due to the rapid growth in population size and increased lobbying from the main farmers’ union in Québec to substantially reduce the number of geese. Despite all the remaining challenges outlined above, the management of this population to date can be considered a success story. We can identify several reasons for that, but one of the key elements was undoubtedly the close collaboration between the CWS and university researchers, which was initiated well before the emergence of the overabundance problem. The implementation of action plans was facilitated by this collaboration, which allowed managers to base their decisions, especially controversial ones such as the spring conservation harvest, on solid scientific grounds. This scientific programme helped in identifying the causes and potential consequences of the population expansion, developing an adaptive management approach (Giroux et al. 1998a), and evaluating the effects of various management actions that were put in place to control this population (Calvert et al. 2007). The population models that were developed based upon these studies (Gauthier and Brault 1998; Gauthier and Reed 2007) guided managers in their decisions and helped them target the measures that were likely to be most effective in reaching their goals. This was especially true with respect to the implementation of a special conservation harvest, which required an amendment to the Convention for the protection of migratory birds in Canada and the U.S. Without such close collaboration, the required legal approval as well as the political and stakeholder support would have been very difficult to secure.

Developing and maintaining good lines of communication among international, national but also regional and local partners is essential to manage a population that migrates over long distances through several political jurisdictions. This ensures that all partners have a shared understanding of all the issues and are working toward a common objective, of controlling an overabundant population. Regular meetings with partners and stakeholders are required to share the most recent, up-to-date information on all relevant issues and especially on newly proposed actions. Our experience as well as the approach described by Tuvendal and Elmberg (2015) proves that it is important to find ways to involve all stakeholders in discussions aimed at defining management goals so that all become part of the consensus and supportive of it.

After two decades of consensus management and relative success at a global level, some difficult issues nonetheless remain, especially with regard to the management of geese at a local level. Despite some successes, most local projects that aimed at maximizing the benefits linked to the presence of greater snow geese while minimizing their impact were short-lived. For example, farmers abandoned their participation in a programme where they were fully compensated to set aside fields for geese because this led to issues of weed invasion in those fields. There could be several reasons for those failures including the need to involve many partners, the lack of local people to lead these initiatives, or the difficulty in finding programmes that could fund those initiatives on a recurrent basis. An additional problem is that successful local projects cannot often be easily exported to other regions due to differences in habitat, hunting or viewing opportunities, and agricultural practices. This level of management is clearly a challenge that lies ahead considering the slow progress up to now, and the inherent obstacles associated with it.

## Conclusions

Considering the adaptability of geese, it is important to stay abreast of potential changes in their behaviour in response to changing environmental conditions and to adapt our monitoring scheme in order to remain effective (Lindenmayer and Likens 2009). Indeed, despite our success in stopping the growth of this population, we must recognize that the equilibrium remains precarious because the greater snow goose is a species that can rapidly and successfully take advantage of changes in its environment. Factors that led to the overabundance in the first place, such as favourable climatic conditions, high food availability, or new habitats, are still present. This poses the risk of renewed population growth at any time, especially in the context of a warming climate (van Oudenhove et al. 2014).
